# Transdiagnostic Network Mapping of Psychopathology in Daily Life: Rationale and Research Protocol

**DOI:** 10.32872/cpe.15939

**Published:** 2025-11-28

**Authors:** Guðrún R. Guðmundsdóttir, Anne Roefs, Alberto Jover Martínez, Anita Jansen, Eiko I. Fried, Esmée Groot, Lotte H. J. M. Lemmens

**Affiliations:** 1 Department of Clinical Psychological Sciences, Maastricht University, Maastricht, the Netherlands; 2 Department of Clinical Psychology, Leiden University, Leiden, the Netherlands; Philipps-University of Marburg, Marburg, Germany

**Keywords:** network approach, mental disorders, ecological momentary assessment, transdiagnostic, individual differences, diagnostic classifications

## Abstract

**Background:**

The burden of mental health problems and the need for more effective interventions is well established. One path towards treatment improvement involves more effective (evidence-based) tailoring, which requires a deeper understanding of differences in individual profiles of psychopathology. The network approach to mental disorders has emerged as a promising framework in this regard, as it sees and assesses psychopathology as individual networks of interacting symptoms and other variables and uses analysis methods that allow fine-grained analyses of (differences in) individual processes.

**Method:**

We describe the protocol of a 6-week ecological momentary assessment (EMA) study in a broad clinical population, designed to capture various transdiagnostic psychopathology relevant states. Participants are Dutch adults (desired = 600) who are currently awaiting intake- or start of treatment for psychopathology. In addition to EMA self-reports, we collect digital phenotyping data, a broad range of baseline data on symptomatology and transdiagnostic traits, and diagnostic classifications after intake. The study’s primary aims are to estimate individual- and group networks of psychopathology (identifying), explore what factors can explain individual differences in networks (linking), and identify potential subgroups based on the networks (clustering). Finally, we plan to evaluate the measures and procedures to facilitate future transdiagnostic EMA (network) research.

**Discussion:**

The prospective study findings have the potential to advance the description, prediction, and assessment of psychopathology and to evaluate the utility of the network framework in achieving these aims. The insights gained may facilitate the evaluation and refinement of current classifications of mental health conditions and alternative transdiagnostic approaches.

## Highlights

- The network approach is a promising way to conceptualise, study and treat mental health problems.
- However, more research is needed to assess its utility, especially related to dynamic individual processes.
- We present a momentary assessment study protocol for network research in a broad clinical sample.
- Key aims involve mapping and comparing transdiagnostic individual- and group-level networks.

Mental health problems are increasingly recognised as a major global concern, with significant personal and societal impacts across health care, the economy, education, and overall well-being (Prince et al., 2007; Walker et al., 2015; Wu et al., 2023). Although much progress has been made in developing effective treatments for various forms of psychopathology, treatment efficacy and high relapse (~60% within 1 year) leave much to be desired (Clark, 2018; Holmes et al., 2018). The modest treatment outcomes have been attributed to a limited understanding of the structure and mechanisms of psychopathology and treatment processes, together with insufficient translation from research to clinical practice (Holmes et al., 2014; Rief et al., 2024). Beyond general mechanisms, a better understanding of individual differences is also needed for adequate treatment tailoring (Wright & Woods, 2020).

Currently, treatment protocols are mostly based on disorder classification categories (e.g., those of the DSM-5; American Psychiatric Association, 2022) which have been shown to consist of very heterogeneous symptom profiles (Allsopp et al., 2019; Fried & Nesse, 2015; Newson et al., 2021). This means that each individual’s profile can be substantially different to that of others sharing the same diagnostic label. Individual profiles also frequently contain characteristics of multiple different disorders (Allsopp et al., 2019; Forbes et al., 2024). Indeed, comorbid diagnoses appear to be the rule rather than the exception (AL-Asadi et al., 2015; Roefs et al., 2022). Yet, much of the evidence used to guide treatment tailoring is based on these categories and often excludes individuals with comorbid diagnoses (Shean, 2014), threatening the representativeness of the populations under study. It follows that classification-informed approaches are likely to be suboptimal for many individuals. These issues highlight the need for more individualised (i.e., idiographic) approaches that transcend diagnostic boundaries (i.e., transdiagnostic) to underpin effective treatments that are tailored to the individual, not the disorder.

A novel and promising framework that has rapidly gained traction in recent years and that may be well-suited to address these challenges is the *network approach to mental disorders* (Borsboom, 2008, 2017). Here, the emphasis is on studying idiosyncratic ‘systems’ of interacting symptoms and other relevant variables rather than typical ‘syndromes’ or disorder categories presumed to share a common (biological) underlying cause (Borsboom, 2017; Bringmann et al., 2022; Fried, 2022). This protocol paper describes a large-scale ecological momentary assessment (EMA) study in a clinical sample embedded within a larger initiative to evaluate the scientific and clinical utility of this approach (Roefs et al., 2022). In the following, we outline the overarching aims and guiding principles of this study and describe the design and its development, followed by a reflection on the project’s strengths and challenges. In doing so, we respond to recent calls for more open and transparent practices in clinical psychology research (Rief et al., 2024) and hope to stimulate and help guide future efforts using EMA and transdiagnostic network approaches in mental health research.

## The Network Approach to Psychopathology

The network approach to mental disorders is often described as a paradigm shift in how psychopathology has long been conceptualised, studied, and practised – as it moves away from the prevailing common-cause and latent-variable models. These latter models see the disorder as explaining its symptoms and assume some underlying, often biological, common cause of these symptoms (Roefs et al., 2022). However, diagnostic categories are descriptive labels, not causal explanations (Borsboom & Cramer, 2013), and despite much research, there is little evidence to suggest that specific mental disorders can be predicted or explained by neurobiological factors (Scull, 2021). Nevertheless, this often termed ‘medical model’ of mental disorders is a prevailing narrative reinforced by leading (mental) health institutions’ causal and disease-laden language (Kajanoja & Valtonen, 2024).

Inspired by a wider framework of complex systems science (e.g., Olthof et al., 2023), the network approach sees the symptoms not as outcomes of the disorder or some common underlying cause. Instead, it sees psychopathology as an emergent and dynamic property arising from the symptom interactions themselves (Borsboom, 2008, 2017; Borsboom & Cramer, 2013). Importantly, recent theoretical advances pose that a ‘symptom’ in this context can be any biopsychosocial element (e.g., emotions, appraisals, behavioural tendencies, contextual factors) that contributes to the development and maintenance of a state of psychopathology, and not only ‘symptoms’ as defined by diagnostic manuals (Fried & Cramer, 2017; Roefs et al., 2022). As such, the network approach is transdiagnostic, seeing each individual’s problem as an idiographic system that may contain elements (i.e., *nodes*) associated with various diagnostic labels.

The application of the network approach to mental disorders has sparked much interest among researchers and practitioners (Kashihara et al., 2025; Roefs et al., 2022), and empirical studies utilising it are accumulating quickly. Initial results are promising but also highlight that much work is still needed for the approach to mature and realise its scientific and clinical potential (for reviews, see Contreras et al., 2019; Kashihara et al., 2025; Robinaugh et al., 2020). Importantly, much of the existing empirical work has been cross-sectional. Albeit informative, cross-sectional networks cannot address the dynamic nature of psychopathology or nuanced individual differences in the dynamic interactions that – according to the network approach – drive the disorder (Borsboom, 2017; Bringmann et al., 2022). Although some work has been done using time-series networks (e.g., Levinson et al., 2022; McGhie & McNally, 2025), these studies have primarily focused on a few specific mental disorders. Therefore, more work is needed using time-series data to uncover within-person networks across the broader psychopathology spectrum.

Some first steps have been taken towards this aim through work carried out by members of the Dutch research consortium New Science of Mental Disorders (NSMD), which was set up to advance the study of psychopathology from the network perspective and in which the current project is embedded. Examination of the network structures among undergraduate students with high vs. low levels of general psychopathology revealed consistent differences in node averages, small differences in the structure of average within-person networks, but considerable heterogeneity in individual networks overall (Jover Martínez et al., 2024a, 2024b). The lack of differences in group-level networks, in contrast to the heterogeneity observed in individual-level networks, aligns with prior work to suggest limited translatability from the group to the individual level of analysis (Levinson et al., 2022; Reeves & Fisher, 2020). Other recent work has also found considerable heterogeneity in networks within the same disorder category (i.e., major depressive disorder), even when analysed separately by severity levels (Ebrahimi et al., 2024). These findings highlight the need to study individual– in addition to group-level processes. Further, we propose to start at the individual level and use bottom-up approaches to identify more homogenous groups of psychopathology profiles that better translate to the individuals within these groups. Such subgroups can help evaluate current classification systems and identify common patterns that can potentially serve as more insightful heuristics in clinical practice and research compared to common classifications. To assess this most adequately and stretch the network *mapping* space to a broader range and severity of psychopathology, the next step involves extending this line of transdiagnostic research to a broad clinical population.

## The Network Mapping Study

### Central Objectives

The overarching aim of the *Network Mapping Study* (NMS) is mapping (individual) networks of psychopathology in a broad clinical population of individuals awaiting treatment. Through this mapping, we seek to achieve the following three central objectives: (1) *identifying* differences across people in individual transdiagnostic networks of psychopathology; (2) *linking* these individual differences to transdiagnostic traits (e.g., self-control), variables in the external field (i.e., contextual factors, such as social support), and disorder classifications (e.g., DSM diagnoses); and (3) *clustering* individuals based on their networks to see whether network similarities can point to meaningful subgroups and whether network-based clusters overlap with common disorder classifications (e.g., DSM). Table 1 presents an overview of example research questions we aim to examine using these data.

**Table 1:** Overview of Research Questions

Objective	Example Research Questions
Identifying	What do individual networks of psychopathology look like in a broad clinical population (i.e., within-person level)? How do the networks differ across individuals (i.e., between-person level)?
Linking	How do between-person differences in the networks relate to differences in scores on standard questionnaires of psychopathology, transdiagnostic traits, and other (e.g., contextual) factors (i.e., mediating or moderating associations)? What are the similarities and differences between network structures of individuals with a similar clinical presentation (e.g., DSM diagnosis)?
Clustering	Can individual networks be used to derive more homogeneous subgroups based on their similarities through clustering methods? How do the identified subgroups differ in terms of their network structures, standard questionnaires of psychopathology, transdiagnostic traits, and other (e.g., contextual) factors? How do the identified subgroups align with common disorder classifications (i.e., DSM diagnoses) and alternative taxonomies of psychopathology (e.g., HiTOP spectra and symptom components; see Kotov et al., 2017)?

Achieving a better understanding of the dynamic interactions of mental health problems in individuals’ daily lives and how individuals differ in these processes is an important step in bridging the network-informed understanding of psychopathology and its potential clinical applications. Beyond evaluating the usefulness for advancing mental health science and research practices, the findings can facilitate potential applications of EMA- and network-derived insights for diagnosis, case conceptualisation and treatment tailoring; and can help evaluate the viability of such approaches in clinical practice.

### Guiding Principles

The main principles guiding our research efforts are four-fold. First, in achieving our primary goal of gaining an overview of what individual networks look like in a wide variety of mental disorders, we strive for both *breadth* – via transdiagnostic measures and a broad range of pathology, and *depth* – via studying idiosyncratic processes. Second, we strive to bridge the *nomothetic* and *idiographic* levels of analysis by studying group– as well as individual networks and focusing both on the trait– and state aspects of psychopathology. We agree with recent calls to distinguish between these levels (DeYoung et al., 2022; Lunansky et al., 2020; Rief et al., 2024) and believe that a holistic picture of psychopathology emerges when the insights gained across levels are integrated. It is clear that processes at the different levels often do not converge (Fisher et al., 2018), not only because of individual differences that get lost when aggregating data but also because different aspects of psychopathology occur at different time scales. Psychopathology is not only a system of daily dynamics in emotions, thoughts and behaviour (i.e., states) but also of a more stable (yet malleable) aspect of the self, reflected in personality characteristics and identity (i.e., traits; Klimstra & Denissen, 2017; Lunansky et al., 2020; Wright & Hopwood, 2022). Thus, we intend to explicitly *link* dynamic state interactions (i.e., captured through momentary assessment) with more stable trait representations of psychopathology (i.e., captured using trait questionnaires). Third, for all analyses of the resulting data, we aim to use state-of-the-art analysis methods, keeping track of the latest developments in network analysis and always striving to use the most optimal and fit-for-purpose methods. This also means considering alternative models (other than networks) in case they turn out to be superior. Importantly, the consortium is devoted to assessing whether there is added value in the network approach, not in *confirming* it. This is directly linked to our fourth principle: to keep sight of the ultimate aim, which is to better explain and predict psychopathology in a way that can be useful for both research and practice, balancing complexity and specificity (*methodological/statistical perspective*) on one hand and practicality and generalisability on the other (*applied/clinical perspective)*. To foster such a balanced approach, the consortium comprises statistical scientists, (clinical) psychological scientists, and clinical practitioners who continually share expertise and perspectives.

## Method

### Design

**Figure 1:**
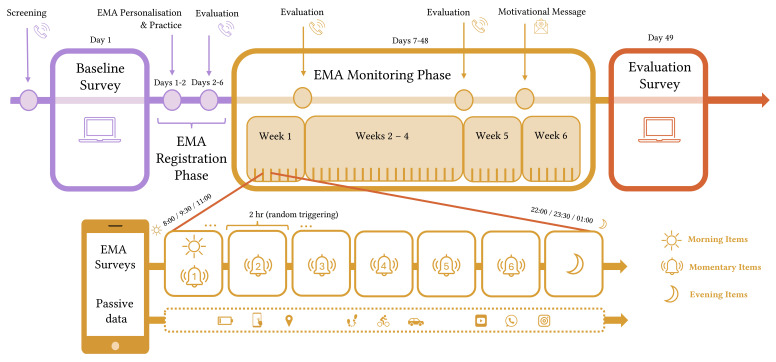
Graphical Overview of the Study Design

Figure 1 depicts a schematic overview of the study design. The study involves a baseline measure and a 6-week ecological momentary assessment (EMA) protocol in a Dutch clinical population with a wide range of psychopathology. The baseline questionnaire intends to provide a comprehensive picture of psychopathology using common measures of mental disorder symptomatology and various transdiagnostic traits. The EMA consists of brief surveys capturing a broad range of state-like symptoms and contextual variables. In addition, passive data is collected from participants’ smartphones during the EMA phase. After the EMA, participants also complete a short evaluation survey asking about their experiences with the study.

The design is largely based on a previous study within the consortium (the *Student Mapping Study*; SMS) specifically developed with transdiagnostic network research in mind Jover Martínez et al., 2024a) and validated in a student population (*n* = 262; Jover Martínez et al., 2025). The EMA battery in the SMS included the full range of psychopathology and was informed by expert opinion collected with a survey and focus groups. Clinicians were queried on the variables most relevant for the mental disorders they specialise in, what transdiagnostic variables they would ask individuals of any disorder and to what extent they believe these variables fluctuate (i.e., momentarily, daily or weekly). We built on and adapted the SMS protocol based on differences in study aims and target groups, insights gained from analyses of the SMS data (e.g., compliance, variabilities in item scores), review of the literature, and follow-up expert meetings among researchers with either methodological or clinical backgrounds (mostly within the consortium).

The project is funded by the Dutch Ministry of Education, Culture and Science (NWO gravitation grant number 024.004.016) and received ethical approval by Maastricht University Ethics Review Committee Psychology and Neuroscience (ERCPN; [nr. 225.97.07.2020]). The study complies with regulations at the University of Maastricht and GDPR guidelines. The entire study protocol was preregistered before data collection started (Guðmundsdóttir et al., 2024S). Data collection is currently ongoing (*n* enrolled at submission = 6).

### Participants and Recruitment

Participants are individuals referred to the adult care units (‘volwassenzorg’; 18-65 years of age) of participating (specialised) mental health care clinics in the Netherlands who are currently awaiting intake- or start of treatment. To participate, individuals need to be proficient in Dutch and own a smartphone. We include the full range of psychopathology and only exclude those who are already receiving treatment elsewhere, those with symptoms that require immediate (crisis) care (e.g., severe suicidality) or that could severely impact the understanding of the EMA items (e.g., symptoms of psychosis). Potentially eligible participants will be informed about the study by the clinics during the registration- and intake phase (through flyers, video, website, information letter, word of mouth). After reading the recruitment materials, individuals express their interest by contacting the research team via phone, email or the project website. After a screening phone call to ensure inclusion and exclusion criteria are met, eligible participants receive a link to the online informed consent form. We will consider additional routes of recruitment if necessary.

### Procedure

Participants first complete the (~80 min) online baseline questionnaire, followed by the EMA phase and, finally, the evaluation survey. The EMA phase starts with a registration in which participants respond to a set of questions that will be used to personalise the EMA. This personalisation involves 1) aligning the triggering of surveys to participants’ waking times and 2) preventing the inclusion of (almost) completely non-applicable items for specific participants (e.g., purging, wish to die), thus reducing unnecessary burden. Participants proceed with one practice day in which they can get familiar with the protocol before data collection starts.

Through the Avicenna smartphone app (Avicenna Research Inc., 2025), participants are prompted to answer surveys seven times per day for a total of 42 days (294 surveys in total). Each day, participants receive three types of EMA surveys triggered at different frequencies: one morning survey, six momentary surveys, and one evening survey. The first measurement of the day includes both the morning survey and the first momentary survey (see Figure 1). The evening survey is triggered separately after the last momentary survey.

The survey notifications are prompted semi-randomly in intervals of 2 hours. For all EMA surveys, participants receive in-app notifications when they are due and reminders when they are about to expire. After the initial prompt, participants have 45 minutes to complete each morning and evening survey but 20 minutes for each momentary survey. Each survey should take about 3-6 minutes to complete, depending on survey type, number of personalised items, and responses to items with skip logic. Participants receive an evaluation phone call after the first and fourth week and a motivational e-mail after the fifth week (in addition to the screening and initial evaluation phone call). To further improve EMA compliance, participants receive reminder e-mails or a phone call when responding deviates from their usual compliance and when responding drops below 50%. Participants are compensated with up to €100 based on compliance with the EMA protocol (€0.33 per EMA survey and €10 for the baseline survey), and those interested can receive a personalised report of their EMA data. During the entire study period, participants can contact researchers if they have questions, and they can withdraw from the study at any time without providing a reason and without any effect on their treatment or waiting time. Waiting time will never be extended for participation, and if treatment starts earlier than anticipated (which we do not expect to occur often considering the standard waiting time and recruitment strategies), participants will, by default, drop out of the study. Participants can continue the monitoring phase at their own wish, but any data collected after the start of treatment will not be used for the primary analyses.

### Measures

In the following, we provide an overview of the measurement battery. A comprehensive overview of all measures, scoring information, translation information and references, and a list of all EMA items in both English and Dutch is available in Guðmundsdóttir et al., 2025S-a).

#### Baseline Measures and Diagnostic Information

The baseline measurement battery consists of demographic and related information and 19 scales measuring psychopathology and transdiagnostic traits. The measures employed are validated scales commonly used to measure symptoms and specific mental health disorders in line with the DSM-5, and wherever possible, validated Dutch translations of these scales. Some scales were translated by the research team (see Guðmundsdóttir et al., 2025S-a). Additionally, we will receive diagnostic and intake information from the participating mental health institutions from which participants are recruited. Table 2 contains an overview of the baseline and diagnostic measures.

**Table 2:** Overview of Baseline Self-Report Measures and Diagnostic Information

Category	Variables
Demographics	Age, gender, nationality
Anthropometrics	Height, weight
Education and employment	Highest achieved level of education, current student status, current employment status
Relationship and children	Current relationship status, number of children
General mental health and treatment	Primary mental health complaint leading to seeking treatment, previous pharmacotherapy, general symptom severity
Measures of specific psychopathology	General depression severity, stress and anxiety, depressive disorder symptoms, attention deficit hyperactivity disorder symptoms, autism spectrum disorder symptoms, disordered eating symptoms, post-traumatic stress symptoms, symptoms of substance use disorders (alcohol and drug use, respectively), obsessive-compulsion disorder symptoms
Transdiagnostic psychopathology	Sexual dysfunction, insomnia severity, levels of personality functioning, dysfunctional personality traits, self-esteem, fear of negative evaluation, dichotomous thinking, self-control, work- and social adjustment, stressful life experiences, trauma
Diagnostic information^a^	Official DSM-5 diagnosis (primary and comorbid classifications), Health of the Nation Outcome Scale (HoNOS+) scores and outcomes, dates of intake, diagnosis, and start of treatment

*Note.* For the full measurement battery, see Guðmundsdóttir et al., 2025S-a).

^a^reported by mental health institutions.

#### EMA Items

Items from the SMS were translated and (when applicable) adapted (e.g., due to differences in target population and language, or to improve clarity), and additional items were formulated by a team of native Dutch speakers. In the morning survey, participants rate sleep quality, nightmares, and how they feel about the upcoming day. The momentary surveys are of central interest for network estimation and capture a broad range of transdiagnostic states relevant to modelling psychopathology as a system. Items include affective states, physical and physiological states, cognitions, cravings, behaviours and interpersonal context that are likely to show sufficient variability within days, which is necessary for network estimation. The evening survey (like the momentary survey) also consists of psychopathology-relevant states, but ones that either concern specific events, have a low daily base rate, or are less likely to fluctuate throughout the day. For example, participants report their perceived social support, deficits in functioning, (urge to) self-harm or harm others and sense of meaning in life. Here, participants are also asked to report in an open format the most unpleasant and pleasant event that day and when it happened. These items can provide important context for interpreting the momentary patterns at both the within- and between-person levels (e.g., what might explain shifts in patterns across days or stable differences across individuals), even if not used as nodes in the (momentary) networks. Most EMA items are answered on a visual analogue scale (VAS; no tick marks but labels on either side [e.g., “very much” and “not at all”] and a dot with an integer between 0 and 100). The VAS format was preferred over the 7-point Likert scale used in the SMS based on recent (currently unpublished) results suggesting that VAS may be better suited for capturing affective states and in the presence of floor or ceiling effects (Haslbeck et al., 2024).

In addition to the self-report surveys and with the informed permission of participants, passive data is also collected from their smartphones via the Avicenna app. This contains information on their device status, location, motion and app usage (see Table 3). It is emphasised to participants that at no point is the content of their text messages, phone calls, social media or other online activity accessible to the researchers. Table 3 contains an overview of the EMA measures, and the full list of items can be found in Guðmundsdóttir et al., 2025S-a.

**Table 3:** Overview of EMA Self-Report and Passive Measures

Category	Variables
Morning	
Sleep	Sleep quality, nightmares
Perceptions about the upcoming day	Nervousness about what could happen during the day, looking forward to the day
Momentary	
Affect states	General negative and positive affect, cheerfulness, sadness, guilt, anxiety, irritability, gloom, loneliness, stress, anger, hopelessness, shame, emptiness*, disgust*
Physical/physiological states	Fatigue, pain or other physical discomfort, nausea*, trembling*, heart palpitations*
Self-satisfaction	Satisfaction with self, satisfaction with physical appearance
Cognition/appraisal	Concentration, worry, intrusive thoughts, specific cravings/urges (e.g., cigarettes, drugs, sex), enjoyment of activity, detachment*
Behaviour	Type of activity engaged in (e.g., work, rest, exercise), giving into/losing control over cravings/urges, avoidance (e.g., thoughts, activities, people), impulsivity, compulsions, body scanning*, scanning environment*
Interpersonal context	Being alone or with others, type of company (e.g., friends, family), enjoyment of company, perceived stress due to company
Evening	
Day reflection	General satisfaction with the day
Cognition/appraisal	Perceived deficits in functioning, perceived social support, perceived meaning in life, wish to die*, urge to self-harm*, urge to harm others*
Behaviour	Self-harm*, aggression*, binge eating*, compensation behaviours*
Daily events	Events perceived as most pleasant and most unpleasant, brief description (open-ended) and timing of these events
Continuous / passive	
Digital phenotyping	Device status (battery status, screen state), location and motion (geolocation, step count and type of activity), frequency and duration of using specific apps ^a^ (e.g., YouTube, Instagram; but only possible for Android users)

*Note.* *Starred variables are only presented to participants who indicated in the registration phase that they apply to them at least some of the time. For the full measurement battery, see Guðmundsdóttir et al., 2025S-a.

^a^No app content is collected (e.g., messages, contact details).

#### Statistical Approaches

The arsenal of network analytic approaches is rapidly growing as the field advances (for a recent overview, see Briganti et al., 2024). For this reason, combined with the exploratory nature of this project, we do not, at this stage, outline in detail or preregister any specific models. Instead, we provide a general summary and examples (see Table 4) for the three central objectives based on the state-of-the-art at the time of writing. For the *identifying,* we plan to estimate individual- and group-level networks using variations of vector autoregressive models (e.g., Bringmann et al., 2013; Epskamp, 2020; Epskamp et al., 2018; Haslbeck & Waldorp, 2020) and assess heterogeneity in individual models (e.g., Hoekstra et al., 2023). For the *linking*, we plan to conduct moderation analyses of the networks (e.g., Haslbeck et al., 2023; Proppert et al., 2025). For the *clustering*, we plan to run data-driven clustering algorithms to derive subgroups based on the networks (e.g., Ernst & Haslbeck, 2025; Gates et al., 2017; Ntekouli et al., 2023; Park et al., 2024). Going beyond the more traditionally used models developed in psychology and psychometrics in the last years, such as the vector autoregressive models mentioned above, we are generally interested in modelling frameworks that can be leveraged to model data as systems, including but not limited to dynamic structural equation modelling, complex dynamic systems modelling, and machine learning.

**Table 4:** Overview of Potential Network Analysis Approaches for the Central Objectives

Objective	Example Analytical Approaches
Identifying	
…differences across people in individual transdiagnostic networks of psychopathology	(Multilevel) Graphical VAR (Bringmann et al., 2013; Epskamp, 2020; Epskamp et al., 2018) Time-Varying Mixed Graphical VAR (Haslbeck et al., 2021; Haslbeck & Waldorp, 2020) Individual network invariance test (INIT; Hoekstra et al., 2023)
Linking	
…individual differences in networks to transdiagnostic traits, variables in the external field and disorder classifications	Group Comparison for Multilevel VAR (Haslbeck et al., 2023) Two-step approaches regressing individual network parameters on predictors (Proppert et al., 2025) Multilevel VAR with continuous moderators of network parameters
Clustering	
…individuals based into subgroups on their networks and assessing whether these subgroups overlap with common disorder classifications	Latent Class VAR, Mixture Multilevel VAR (LCVAR; MMVAR; Ernst et al., 2020, 2024; Ernst & Haslbeck, 2025) Chain Graphical VAR (Park et al., 2024) Group Iterative Multiple Model Estimation (GIMME; Gates et al., 2017) Model-based approaches using (non-)linear machine learning models (Ntekouli et al., 2023) Individual Network Invariance Test (INIT; Hoekstra et al., 2023) to assess network heterogeneity within and across clusters Regressing cluster membership on predictors

*Note.* The examples presented here are approaches that can be utilised for the three central objectives – based on the state-of-the-art at the time of writing. We will keep track of the latest developments in the field and always strive to use the most optimal methods. We are also interested in exploring how the data can be leveraged within other modelling frameworks.

## Study Potential and Challenges

The current project has some noteworthy potential, both within and beyond the network approach. Its strengths lie in the transdiagnostic focus, the sampling from a clinical population with a broad range of psychopathology, and the adoption of state-of-the-art methodology and complex systems thinking. As far as we are aware, this is one of the largest transdiagnostic EMA studies in a clinical population to date. Of course, a project like this also faces several important challenges.

In a sea of relevant constructs and measures, selecting the final measurement battery is difficult, particularly when the goal is to map a wide range of psychopathology. Aside from including a representative set of items, we need to consider participant burden, compliance, response biases, and the timescale with which the variables fluctuate. For this project, these challenges were addressed by combining multiple sources of information – both qualitative and quantitative. The initial protocol was based on clinical experts’ views collected through focus groups and survey data, and then refined based on insights from a validation study in a student sample (Jover Martínez et al., 2025), differences in population and study goals and a review of the literature. As always, there is room for improvement, but one of the values of this project involves the insights gained (e.g., via participant feedback and psychometric analyses) that can help further refine and adapt this and similar protocols for future transdiagnostic (network) research.

A measurement challenge lies in determining the amount of data needed that balances reliable and well-powered analyses on the one hand and participant burden on the other. There are no clear guidelines on how many observations are needed to estimate individual networks, but early simulation work suggests that small networks (six variables) can be reliably estimated with approximately 100 observations (Mansueto et al., 2023). Based on that, the current studies should provide adequate reliability and statistical power despite missingness. Note, however, that reliability and power are dependent on various other factors, such as network- and item characteristics (e.g., number of nodes, network density, item variability, type of missingness) and whether group- (multilevel) or individual networks are estimated. Thus, any network estimation using these data needs to be made with an eye to such factors. Although more observations are always desirable, a longer EMA phase would not be feasible given the study context (i.e., waitlist for clinical intervention) and analysis aims (i.e., many classic network estimation techniques rely on the assumption of stationarity, which is more likely to be violated for longer timespans; Jordan et al., 2020). Further, a more intense scheduling procedure would likely lead to worse compliance (Eisele et al., 2022; Vachon et al., 2019).

We also foresee challenges on the analysis front. For example, there are concerns (both methodological and conceptual) attached to combining variables at different ‘levels’ (de Boer et al., 2021; Wichers et al., 2021), whether it be different time scales (e.g., momentary vs daily, state vs trait), data types (e.g., self-report vs passive data, internal vs external factors), or construct breadth (e.g., discrete emotional states vs broad meta-constructs). Current network models for EMA data cannot easily incorporate different data types and variables that fluctuate at different rates (Bringmann, 2024), but this may become possible with methodological advancements (e.g., through ‘multi-layered’ networks; Riese & Wichers, 2021). Until then, we advocate caution in estimating and interpreting networks with different types of nodes, starting with more homogenous self-report elements measured at the momentary level, which this study is specifically designed for.

Another analytical challenge is that if the goal is to explore inter-individual differences and subgroups, all networks must contain the same nodes. However, the relevance and fluctuations of nodes likely differ across individuals. To tackle this, we plan to select a generally representative set of nodes for individual comparisons and consider alternative nodes for specific subgroups or when exploring individual networks. This highlights again the need to consider both the nomothetic and idiographic levels of analysis, one of our central objectives.

## Conclusion

The *Network Mapping Study* is a research initiative primarily conducted to expand current knowledge on the structure and dynamics of psychopathology and evaluate the utility of the network approach. In addition to the clinical population, its key strengths lie in the transdiagnostic measurement battery and rich information on individuals’ psychopathology at both the trait and state levels, providing ample opportunity for collaboration and exploring novel research questions. The amount and richness of the data should lend themselves well to various types of analyses, addressing the nuances of psychopathology from different angles and approaches. When combined with evidence from experimental and intervention studies and work within other (transdiagnostic) frameworks, the insights gained may be integrated into larger knowledge structures that can help move the field forward and achieve the urgent goal of improving measurement, classification and personalised treatment of mental health problems.

## Supplementary Materials

The Supplementary Materials contain the following items:

- the preregistration for the study (Guðmundsdóttir et al., 2024S)
- the full ecological momentary assessment battery in both Dutch and English (Guðmundsdóttir et al., 2025S-a)
- the project's OSF repository (Guðmundsdóttir et al., 2025S-b)

GuðmundsdóttirG. R.
RoefsA.
GrootE.
Jover MartínezA.
JansenA.
LemmensL. H. J. M.
 (2024S). Network mapping of psychopathology: Estimation of individual and group networks in a clinical sample
[Preregistration]. PsychOpen. https://osf.io/93cwz


GuðmundsdóttirG. R.
RoefsA.
GrootE.
Jover MartínezA.
JansenA.
FriedE. I.
LemmensL. H. J. M.
 (2025S-a). Supplementary materials to "Transdiagnostic network mapping of psychopathology in daily life: Rationale and research protocol"
[Measurement]. PsychOpen. https://osf.io/w4sef
10.32872/cpe.15939PMC1292319341725699

GuðmundsdóttirG. R.
RoefsA.
GrootE.
Jover MartínezA.
JansenA.
FriedE. I.
LemmensL. H. J. M.
 (2025S-b). The Network Mapping Study
[Project repository]. PsychOpen. https://osf.io/keth3


## Data Availability

The project's OSF repository (https://osf.io/keth3/) contains a comprehensive overview of the study measures, including the full ecological momentary assessment battery in both Dutch and English (see also Supplementary Materials accompanying this publication). Relevant materials, including procedures on personalised reports, analysis code, and publications, will be uploaded to this repository. The consortium is currently developing a data-sharing policy with the goal of fostering open collaboration and data sharing.

## Social Media Accounts

*Guðrún R. Guðmundsdóttir:* Bluesky, LinkedIn, *Alberto Jover Martínez:* LinkedIn, *Lotte H. J. M. Lemmens:* LinkedIn, *Anne Roefs:* LinkedIn, *Eiko I. Fried:* X

## Reporting Guidelines

We follow the JARS-Quant (Reporting Standards for Studies Using No Experimental Manipulation).

## Preregistration

The study is preregistered (https://osf.io/93cwz).
